# Freshwater Microbial Eukaryotic Core Communities, Open-Water and Under-Ice Specialists in Southern Victoria Island Lakes (Ekaluktutiak, NU, Canada)

**DOI:** 10.3389/fmicb.2021.786094

**Published:** 2022-02-11

**Authors:** Marianne Potvin, Milla Rautio, Connie Lovejoy

**Affiliations:** ^1^Département de Biologie, Québec Océan, and Institut Intégrative et des Systèmes (IBIS), Université Laval, Quebec, QC, Canada; ^2^Département des Sciences Fondamentales, Université du Québec à Chicoutimi, Saguenay, QC, Canada; ^3^Groupe de Recherche Interuniversitaire de Limnologie (GRIL), Montreal, QC, Canada; ^4^Center D’Études Nordiques (CEN), Quebec, QC, Canada

**Keywords:** Arctic, chrysophytes, cryptophytes, season, ciliates

## Abstract

Across much of the Arctic, lakes and ponds dominate the landscape. Starting in late September, the lakes are covered in ice, with ice persisting well into June or early July. In summer, the lakes are highly productive, supporting waterfowl and fish populations. However, little is known about the diversity and ecology of microscopic life in the lakes that influence biogeochemical cycles and contribute to ecosystem services. Even less is known about the prevalence of species that are characteristic of the seasons or whether some species persist year-round under both ice cover and summer open-water conditions. To begin to address these knowledge gaps, we sampled 10 morphometrically diverse lakes in the region of Ekaluktutiak (Cambridge Bay), on southern Victoria Island (NU, Canada). We focused on Greiner Lake, the lakes connected to it, isolated ponds, and two nearby larger lakes outside the Greiner watershed. The largest lakes sampled were Tahiryuaq (Ferguson Lake) and the nearby Spawning Lake, which support commercial sea-run Arctic char (*Salvelinus alpinus*) fisheries. Samples for nucleic acids were collected from the lakes along with limnological metadata. Microbial eukaryotes were identified with high-throughput amplicon sequencing targeting the V4 region of the 18S rRNA gene. Ciliates, dinoflagellates, chrysophytes, and cryptophytes dominated the lake assemblages. A Bray–Curtis dissimilarity matrix separated communities into under-ice and open-water clusters, with additional separation by superficial lake area. In all, 133 operational taxonomic units (OTUs) occurred either in all under-ice or all open-water samples and were considered “core” microbial species or ecotypes. These were further characterized as seasonal indicators. Ten of the OTUs were characteristic of all lakes and all seasons sampled. Eight of these were cryptophytes, suggesting diverse functional capacity within the lineage. The core open-water indicators were mostly chrysophytes, with a few ciliates and uncharacterized Cercozoa, suggesting that summer communities are mixotrophic with contributions by heterotrophic taxa. The core under-ice indicators included a dozen ciliates along with chrysophytes, cryptomonads, and dinoflagellates, indicating a more heterotrophic community augmented by mixotrophic taxa in winter.

## Introduction

The Arctic is home to emblematic endemic species that have evolved in response to seasonal isolation and an extreme environment, where light and temperatures are limiting for much of the year. Across the Arctic, lakes and ponds dominate the landscape. The thousands of lakes of southern Victoria Island, Nunavut, are an integral component of local indigenous communities that depend upon them for valuable ecosystem services, providing potable water, food security, and cultural grounding (White et al., 2007). This cultural and ecological intertwining is reflected in the local name, Ekaluktutiak, for the main hamlet (Cambridge Bay) and the surrounding region of southern Victoria Island, which means good fishing place and refers to the abundance of both lake trout (*Salvelinus namaycush*) and Arctic char (*Salvelinus alpinus*) (Grosbois et al., 2022). The aquatic macrofauna in the lakes (zooplankton and emergent insects) that support the higher food webs are in turn largely dependent on small single-celled microbial eukaryotes (phytoplankton and other protists) that live in the lakes and ponds. These protists are abundant over the short summer, when surface waters are exposed to 24 h of light and reach temperatures into the teens well above freezing (Rautio et al., 2011a). By late September, the lakes are covered in ice and the underlying waters near the freezing point. The lake ice typically persists well into June or early July (Imbeau et al., 2021). However, despite dark and cold conditions, life continues throughout the winter (Hampton et al., 2017; Schneider et al., 2017; Grosbois and Rautio, 2018).

The Arctic is experiencing rapid warming, and the duration and phenology of the ice-free season is becoming less predictable, with the observed rates of climate-driven change in the Arctic exceeding existing climate model scenarios [Arctic Monitoring and Assessment programe [AMAP], 2017; Meredith et al., 2019]. The lakes are becoming increasingly subjected to longer ice-free periods as northern Canada warms at up to 5 times the rate of the global average (Meredith et al., 2019). The ecological function or health and the biodiversity of Arctic freshwaters are likely to be impacted by the changing conditions (Wrona et al., 2006). However, little is known about the microbial species that underpin the lake ecosystems, which contribute disproportionately to Arctic and sub-Arctic biodiversity (Rautio et al., 2011a; Charvet et al., 2012a).

Seasonal comparisons between ice-covered and summer open-water conditions in Arctic freshwaters are rare (Vigneron et al., 2019), impeding basic understanding of ecosystem function and possible vulnerability. The lack of data on the seasonality of microbial eukaryotes extends to Victoria Island, Nunavut, particularly at the level of species or putative ecotypes, which can now be distinguished using molecular techniques. As part of a larger study on the overall connectivity of Ekaluktutiak (Cambridge Bay) lakes, we carried out a survey of 10 diverse lakes with the aim of identifying ubiquitous protist species that would constitute the microbial eukaryote contribution to the core microbiome of the region. The area is accessible from the Canadian High Arctic Research Station (CHARS), which is set to become a monitoring site [Environmental Research Area (ERA), designated the CHARS-ERA] with a mandate to document ongoing change in the Arctic. Knowledge of the makeup of this core community can be used to monitor change and identify major perturbations in the future.

Specifically, we targeted a range of morphometrically diverse lakes in the CHARS-ERA Ekaluktutiak area. This region includes Greiner Lake, which is of particular importance to the local community of Cambridge Bay. For this study, sampling sites were from the same watershed and included lakes connected to Greiner Lake and hydrologically isolated ponds. In addition, two larger lakes outside of the Greiner watershed, Tahiryuaq (Ferguson Lake) and Spawning Lake, were also sampled to increase limnological diversity. These larger lakes are extensively used by sea-run Arctic char (Moore et al., 2016; Harris et al., 2020). We then further compared under-ice microbial eukaryotic plankton with open-water plankton in most of the same lakes, including Greiner Lake.

Water samples for nucleic acids were collected along with limnological metadata with the aim to identify relevant physical and chemical variables associated with total community compositions in the different seasons and lakes. We then focused on commonalities among the lakes by distinguishing a “core” community of microbial eukaryotes in the lakes with a view toward laying the groundwork for future studies. The concept of core in this case was firstly based on universal occurrence in all lakes sampled either under open-water or ice-covered conditions. In addition to presence/absence, the core community needed to meet the other criteria suggested by Shade and Handelsman (2012), which are persistence, connectivity, community composition, and phylogeny. The connectivity was based on geography, with the core taxa being found in lakes that were all within 40 km of Greiner Lake. Persistence was based on these core taxa occurring either under ice or in open waters of Greiner Lake over 3 years. The other criteria of Shade and Handelsman (2012), community composition and phylogeny, were implicit in our approach using molecular identification. To this end, the microbial eukaryotic communities were identified using high-throughput amplicon sequencing targeting the V4 region of the 18S rRNA gene (rDNA) and 18S rRNA (rRNA). The core operational taxonomic units (OTUs with 98% similarity) consisted of OTUs found in all open-water or all under-ice samples, a subset of which were OTUs that were found in all the sampled lakes irrespective of season. We then identified the seasonal specialists and indicators for open-water (summer) and under-ice (winter and spring) conditions.

## Materials and Methods

### Sampling and Laboratory Analysis

**FIGURE 1:**
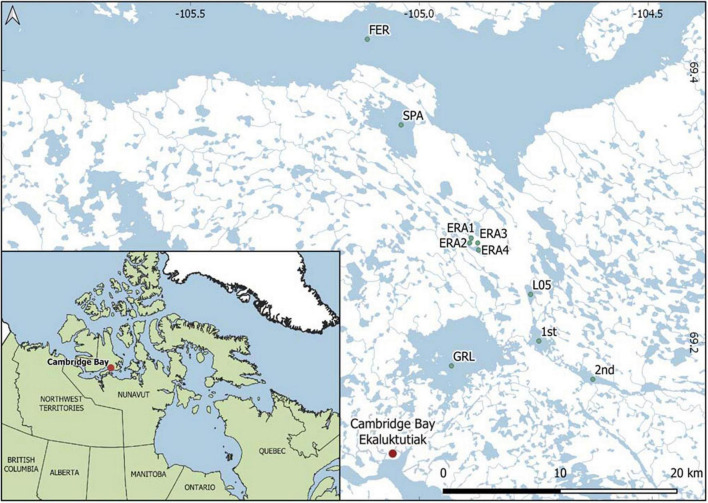
Map of the study area showing the lakes sampled. Lakes with names are Tahiryuaq, previously known as Ferguson (*FER*), Spawning (*SPA*), Greiner (*GRL*), and Inuhuktok (*L05*). Map was created using Q-GIS 3.14 based on Landsat 8 Operational Land Imager (OLI) imagery and *in situ* GPS data.

The sampling sites were in the CHARS-ERA located on Victoria Island, which is at the western entrance of the Northwest Passage through the Canadian Arctic Archipelago (Figure 1). We sampled a total of 10 lakes over multiple years from June 2015 to August 2018. The lakes were classified by size based on surface area. ERA1–ERA4 were defined as small lakes with areas of 0.75 km^2^ (ERA4), 0.19 km^2^ (ERA1), and 30–40 m^2^ (ERA2 and ERA3). The lakes designated as 1st, 2nd, and L05 (locally known as Inuhuktok) were medium lakes (3.16, 2.68, and 1.11 km^2^, respectively). Large lakes were Greiner (36.9 km^2^), Spawning (12.5 km^2^), and Tahiryuaq (Ferguson Lake, 588 km^2^). Eight of the lakes were from the Greiner watershed, while Spawning and Tahiryuaq were nearby, but in a separate watershed. Spawning Lake drains into Tahiryuaq, which is directly linked to the sea *via* the Ekalluk River. The river flows into Wellington Bay, which is contiguous with Dease Strait and the Arctic Ocean. All the lakes in this region are ice-free for about 3 months of the year, between early to mid-July and the end of September. The open-water samples were collected in September (2015 and 2016) and August (2017 and 2018). The under-ice samples were collected in June 2015, October and November 2017, and April 2018. Samples were collected from the center or the deepest portion of the lake based on soundings for the small lakes and available bathymetry for the larger lakes. Lake water was accessed either by drilling a 254 mm hole through the ice in winter with a Jiffy ice augur or by an inflatable boat or float plane during the open-water season. Water for all lakes was collected using a 2-L Limnos water sampler (Limnos Ltd., Komorow, Poland), which was closed just below the surface. Water from multiple casts was collected and mixed in a larger cleaned container that had been rinsed 3 times prior to filling. In 2015, additional nucleic acid samples from 4 and 8 m were collected from Greiner Lake following the same protocol used for surface samples.

Temperature and conductivity were measured using a Ruskin RBR Concerto probe (Ottawa, ON, Canada). Water for ancillary variables was collected from just below the surface in conjunction with nucleic acid sampling. For chlorophyll *a* (Chl *a*), 500 ml was transferred immediately into separate opaque polycarbonate (PC) bottles. Aliquots of sample water for total nitrogen (TN) and phosphorus (TP) were transferred into acid-washed glass bottles. All water samples were placed directly into a cooler and transported by float plane (Spawning and Ferguson Lakes), helicopter, snowmobile, or all-terrain vehicles to the CHARS campus for filtration and laboratory manipulations within 1–3 h of collection. The samples for Chl *a* were filtered onto Whatman GF/F glass fiber filters, wrapped in an aluminum foil, and frozen at −20°C until extraction in 90% ethanol and spectrofluorometric analysis (Nusch, 1980). Samples for dissolved organic carbon (DOC) were filtered through a pre-combusted Whatman GF/F and stored in acid-washed glass bottles. TN, TP, and DOC samples were analyzed by Environment and Climate Change Canada at the National Laboratory for Environmental Testing (Burlington, ON, Canada) following internal protocols (Environment Canada, 2019).

### Filtration and Laboratory Treatment of Nucleic Acids

Up to 2 L of water for nucleic acids was poured through a 50-μm net to remove zooplankton and then filtered successively through 3-μm pore size 47-mm PC membrane filters and 0.2-μm pore size Sterivex filter units (Millipore, Burlington, MA, United States) using a peristaltic pump system. The 47-mm filters were placed in 1.5-ml microfuge tubes filled with RNAlater; 1.8 ml of RNAlater was also added to the Sterivex units. The preserved samples were initially kept at −20°C until they were shipped south within 3 months, then at −80°C until nucleic acid extraction. All collected (47 mm and Sterivex) filters were extracted separately for nucleic acids using AllPrep DNA/RNA Mini Kit (QIAGEN, Hilden, Germany). RNA was converted to cDNA using the Applied Biosystems High-Capacity cDNA Reverse Transcription Kit (Thermo Fisher Scientific, Waltham, MA, United States). The DNA or cDNA (from RNA) was amplified following protocols and using the V4 primers given in Kalenitchenko et al. (2019). Illumina Mi-Seq sequencing was carried at the Université Laval sequencing facility (Plateforme d’analyze génomique, IBIS). Raw sequences are archived in NCBI Sequence Read Archive (SRA) under the Bioproject accession PRJNA623385 and in EMBL European Nucleotide Archive (ENA) under the accession number PRJEB24089.

### Bioinformatics Analysis

Since the goal of this study was to identify common OTUs from all sampled lakes in the geographical area, we pooled and analyzed all sequencing results to obtain a single OTU table. These included all reads generated from both DNA and cDNA (RNA) templates and from the separate large (3-μm pore size filter) and small (0.22-μm pore size filter) fractions. The two size fractions were collected and sequenced separately to reduce primer bias as the small fraction enriches for smaller cells with low rRNA gene copy numbers that tend to be swamped by larger cells when not size fractionated. Sequence analysis followed the method of Kalenitchenko et al. (2019), with minor modifications. Briefly, read pairs were merged using BBMerge v37.36 (Bushnell et al., 2017), followed by quality filtering with vsearch (maxEE parameter of 0.5) (Rognes et al., 2016). Unique sequences were selected to decrease the computational need for the chimera checking with USEARCH [unoise3, min size = 4 (zotus)] and OTU clustering in USEARCH (Edgar, 2010). A similarity threshold of 98% was used to define OTUs, and the centroid of each OTU was selected for downstream sequence analysis. We then assigned taxonomy using the Wang method in mothur v1.39 (Schloss, 2020), and the PR^2^ database v4.11.0 (Guillou et al., 2013) was used as a reference database. In addition, we performed the same analysis using the Silva v132 database (Quast et al., 2013). An OTU table was then constructed, which contains the number of copies of each unique sequence of OTUs from all separate sample libraries. The final OTU table was then filtered to include only microbial eukaryotes, excluding fungi (essentially chytrids, Cryptomycota and Microsporidiomycota). OTUs matching bacteria, archaea, and multicellular organisms (metazoan and land plants) were also removed. Data from a single geographic location (lake) and unique sampling date were combined to obtain single-site/single-date libraries that were then treated as sample units for downstream analysis (Supplementary Table 1).

### Phylogenetic Analysis

PR^2^ and SILVA taxonomic assignments for individual OTUs concurred for most of the OTUs. When there was disagreement, we carried out individual BLAST searches of the OTUs and assigned them to the closest species-level match, which, in most cases, corresponded to the PR^2^ assignment. Because of the high level of ambiguity in the case of cryptophytes and chrysophytes, we carried out separate alignments using Evolutionary Placement Algorithm (Stamatakis, 2014). We first constructed reference trees containing nearly full-length 18S rRNA gene reference sequences for the cryptophytes and chrysophytes mined from GenBank. Sequences were aligned with the multiple sequence alignment program (MAFFT) using default parameters (Katoh and Standley, 2013). The resulting alignments were trimmed to remove gaps at the beginning and the end of the alignments. The representative sequences of the selected OTUs were mapped onto the reference trees in MAFFT-add (–addfragments, Auto) using the evolutionary placement algorithm within RAxML (GTRGAMMA). The sequences of the representative OTUs were placed on the tree at the node that had the highest likelihood result. Trees were visualized and edited using FigTree v1.4.3. For all the microbial eukaryote OTUs, when more than one OTU (98% similarity) was associated with a single species, separate OTUs were considered as distinct genetic populations since several separated into open-water or under-ice distributions, suggesting ecotypes.

### Statistical Analysis

Statistical analyses were performed using the “vegan” package in R (R Core Team, 2017). We used Hellinger transformed raw (not rarefied) and the complete OTU table to construct a matrix for the Bray–Curtis dissimilarity analysis. A resulting dendrogram was plotted using Ward clustering. Groups defined by the Bray–Curtis analysis were used to run analysis of similarities (ANOSIM) of the samples and similarity percentage (SIMPER) tests.

To address the environmental context behind whole community clustering, we used the vegan package in R to link environmental data (Table 1) with the eukaryote communities assessed by amplicon sequencing. We used the Hellinger-transformed OTU table, raw environmental data, and the Bray–Curtis dissimilarity distance to perform distance-based redundancy analysis (db-RDA) on the complete and on the seasonal subsets of samples (open water and under ice).

**TABLE 1:** Sample environmental data.

Sample	Ice (cm)	DOC (mg L^–1^)	TN (μg L^–1^)	TP (μg L^–1^)	Temp. (°C)	Cond (μ S cm^–1^)	Chl *a* (μg L^–1^)	DL (h)	Size	Group
GRL_23AP18	155	5.6	600	11	1.86	608	0.39	17.23	L	Ice1
L05_24AP18	170	3.8	381	8.3	0.79	382	0.27	17.39	M	Ice1
1ST_26AP18	180	5.2	511	13.1	0.71	496	4.20	17.71	M	Ice1
2ND_26AP18	175	5.1	537	15.5	0.54	440	2.46	17.71	M	Ice1
GRL_10JN15	205	5.7	nd	6.1	1.80	675	1.07	24.00	L	Ice1
GRL_15JN15	195	4.9	nd	7.5	nd	nd	0.86	24.00	L	Ice1
GRL_12JN15	200	nd	nd	nd	nd	nd	Nd	24.00	L	Ice1
ERA4_24AP18	167	8.7	736	8.5	1.14	878	0.10	17.39	S	Ice2
L05_02NO17	40	3.7	333	nd	0.56	318	0.54	6.50	M	Ice2
ERA4_03NO17	44	6.6	661	8.4	nd	nd	0.84	6.35	S	Ice2
2ND_01NO17	36	4.4	456	13.6	0.83	269	1.64	6.66	M	Ice2
1ST_31OC17	40	4.5	350	17.9	0.45	317	2.81	6.81	M	Ice2
GRL_30OC17	25	3.9	384	16.8	2.10	325	0.43	6.96	L	Ice2
ERA1_04NO17	42	8.1	887	13.9	2.80	1,049	2.80	6.19	S	Ice2
ERA1_24AP18	170	15.5	1,493	15.3	0.51	2,195	5.52	17.39	S	Ice2
ERA1_10AU17	0	6.7	560	<5.0	10.80	820	1.60	18.60	S	Open3
ERA3_10AU17	0	7	570	<5.0	10.60	770	3.26	18.60	S	Open3
ERA1_12AU18	0	6.9	493	9.2	9.60	524	2.11	18.30	S	Open3
ERA4_10AU17	0	5.9	425	<5.0	11.10	501	1.15	18.60	S	Open3
ERA4_12AU18	0	6	436	5.7	9.10	818	1.16	18.30	S	Open3
1ST_09AU18	0	4.2	331	5.4	10.00	262	2.57	18.82	M	Open3
ERA2_10AU17	0	15.2	1,400	8	10.80	648	1.28	18.6	S	Open3
GRL_04SE16	0	nd	nd	nd	5.40	344	Nd	14.06	L	Open4
SPA_13AU17	0	3.2	271	<5.0	12.40	295	0.85	18.10	L	Open4
GRL_07AU18	0	6.1	832	8.4	9.50	270	3.14	19.17	L	Open4
FER_13AU17	0	2.1	168	<5.0	8.50	245	1.80	18.10	L	Open4
GRL_09SE15	0	3.2	372	5.2	12.40	266	1.29	14.17	L	Open4
GRL_23AU16	0	3.5	427	6.4	10.80	330	Nd	16.49	L	Open4
GRL_09AU17	0	3.7	298	<5.0	10.80	278	1.71	18.77	L	Open4

*Sample name consists of the lake abbreviation from Figure 1 and the day, month, and year of sampling (JN refers to June).*

*When values were under the limit of detection, half of the detection limit value was used for statistics.*

**FIGURE 2:**
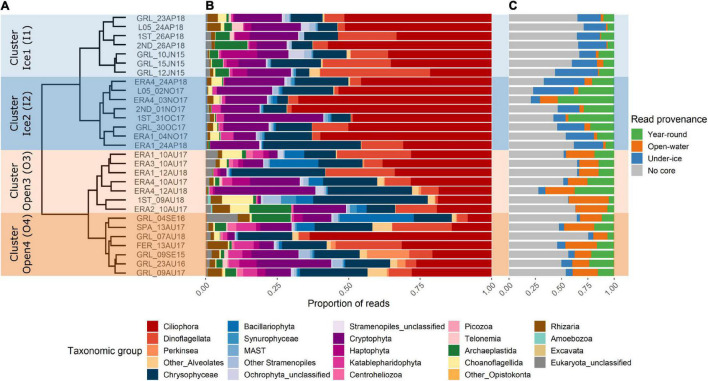
Overall eukaryote diversity in Ekaluktutiak (Cambridge Bay region) revealed with high-throughput sequencing. **(A)** Dendrogram of Bray–Curtis dissimilarity from the Hellinger-transformed operational taxonomic unit (OTU) table containing all protist OTUs. The samples clustered primarily as under ice (Ice1 and Ice2) and open water (Open3 and Open4). **(B)** Relative abundance of reads classified by higher-level taxonomy. **(C)** Relative abundance of reads from the samples within the OTU categories following the initial core definitions. OTUs present in all samples are shown in *green*, OTUs with 100% prevalence in under-ice samples are in *blue*, OTUs with 100% prevalence in open-water samples are in *orange*, and the prevalence of reads from OTUs that were not defined as core is in *gray*.

*Lake order follows the Bray–Curtis group clustering from Figure 2, with (group) Ice1, Ice2, Open3, and Open4 indicated.*

*The detection limit for TP was 0.5 μg L^–1^.*

*DOC, dissolved organic carbon; TP, total phosphorus; TN, total nitrogen; Ice, ice thickness; Temp, temperature; Cond, conductivity; Chl a, chlorophyll a; DL, day length; Size, lake size category; L, large; M, medium; S, small (as defined in text); nd, no data available.*

### Defining the Microbial Eukaryote Core Community

The eukaryotic core OTUs comprise those in all “under-ice” samples and those in all “open-water” samples. We also noted a “year-round” category that contained OTUs that were present in all of the samples in the study. Following the definition of prevalence given in Shade and Handelsman (2012), the under-ice category contained OTUs that were present in all the colder season samples (prevalence under ice = 100%). The open-water category (prevalence open water = 100%) contained all the OTUs that were present in all the open-water samples. We note that the open-water and under-ice core OTUs were rarely exclusive to a particular season, with under-ice core OTUs found in the open-water samples and *vice versa*.

Indicator species were investigated using the IndVal command in R (labdsv package). For the IndVal test, the raw OTU table was converted into proportions. As IndVal compares two groups at a time, the comparison groups were based on the two main clusters from the Bray–Curtis dissimilarity analysis. The significance of the indicator OTUs was set at a *p*-value threshold of 0.05 from 10,000 iterations. The same Bray–Curtis sample clusters were compared to identify OTUs as specialists or generalists with the multinomial species classification method (CLAM). This test classifies OTUs as generalists, specialists of a group, or too rare to be defined.

## Results

### Diversity

Overall, our analysis included 29 one-site/one-date samples from the 10 lakes (Figure 1). A total of 2,717,541 reads were retained after quality and taxonomic filtering, with an average of 93,708 reads per sample (Supplementary Table 1). The filtered and collapsed final OTU table used for all the community analyses (29 samples) resulted in 1,435 OTUs at 98% similarity. The “top five” OTUs, defined as those that were most abundant in terms of read counts in each sample, varied with some overlaps among lakes (Supplementary Figure 1). The *Fasta* files for these and the other OTUs mentioned in the main text and figures are provided in as a Supplementary Data File 1. The five most abundant OTUs in each sample (Supplementary Figure 1) accounted for 19.9–56.2% of the reads in their respective samples. Many of the OTUs in the top five were dominant in more than one lake, with a total of 70 distinct top five OTUs at the 98% similarity level. Alveolates accounted for 39 of these, with 29 ciliates, 9 dinoflagellates, and 1 Perkinsida (Supplementary Figure 1). The other predominant top five OTUs included chrysophytes and cryptophytes, along with one Thraustochytriaceae, one Katablepharida, and two Chlorophyta (Supplementary Figure 1).

At the level of major groups, Ciliophora, Dinoflagellata, Chrysophyceae, and Cryptophyceae were predominant in all samples (Figures 2A,B). However, there were major differences at the OTU level, and the Bray–Curtis dissimilarity analysis revealed that samples clustered first by season, with under-ice samples collected in October, November, April, and June separated from open-water samples collected in August and September (Figure 2). The relative proportions of reads belonging to the different core categories (Figure 2C under ice and open water) followed the clustering pattern of the whole communities. All communities included generalist “all-year” OTUs and non-core OTUs (Figure 2C).

The under-ice samples were more generally characterized by higher proportions of dinoflagellate and ciliate reads, with approx. 40–70% of the reads per sample. This under-ice cluster was further separated into two distinct groups (Figure 2, left hand labels), designated as Ice1 and Ice2. The Ice1 samples collected in spring (April and June) included samples from Greiner Lake (GRL) and from the medium-sized lakes, with slightly higher proportions of Archaeplastida (green algae) and Haptophyta reads; on the other hand, the Ice2 cluster had samples that were mostly from October and November, but also the April samples from smaller lakes. This Ice2 cluster consisted of slightly higher proportions of choanoflagellates and katablepharids, which are heterotrophic flagellates.

The open-water main cluster from the Bray–Curtis dendrogram contained all the samples that were collected from August and September. These were also separated into two sub-clusters: Open3 and Open4 (Figure 2). Open3 included the larger lakes (Greiner, Tahiryuaq, and Spawning), while the Open4 sub-cluster consisted of the small- and intermediate-sized lakes (1st lake and ERA1–ERA4). Open-water samples from L05 and 2nd lake were not available.

The dissimilarity of the four clusters was also significant according to the ANOSIM results. A first statistical test was performed by computing ANOSIM comparing the seasonal clusters (open water *vs*. under ice), which showed significant dissimilarity (*R* = 0.7672, *p* = 0.001). The same statistic was then performed considering the 4 different clusters from the Bray–Curtis cluster analysis, which showed significant dissimilarity among the four sub-clusters (*R* = 0.8008, *p* = 0.001). In summary, dissimilarity of the eukaryote community was primarily determined by the sampling season (open water *vs*. under ice), and there were also significant differences associated with the lake size classes for both seasons.

### Linking Communities to Environmental Data

**FIGURE 3:**
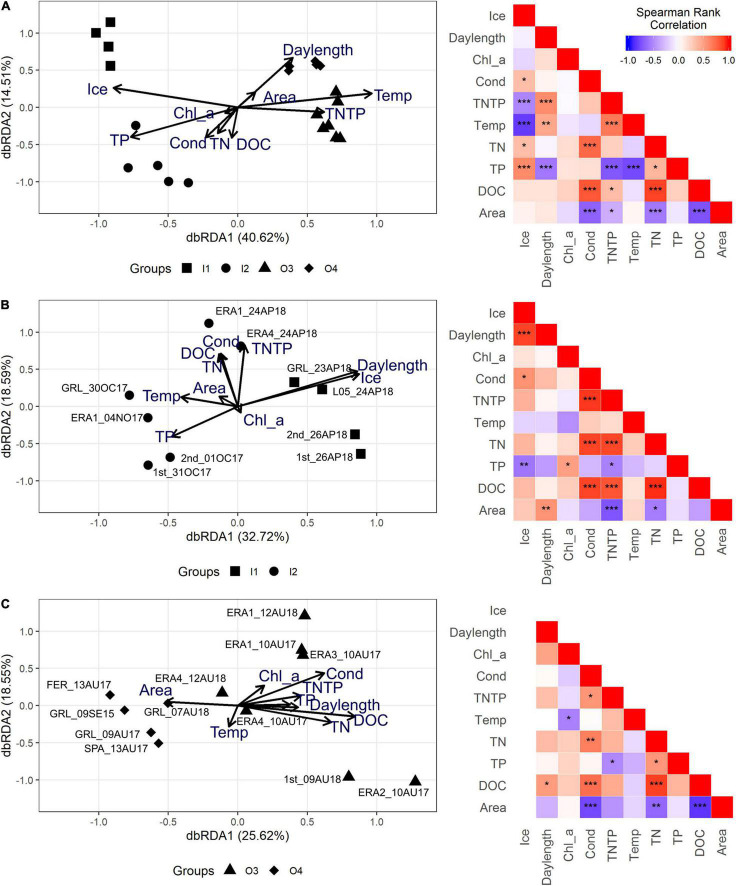
Bray–Curtis distance of the Hellinger-transformed operational taxonomic unit (OTU) table analyzed with different environmental variables. On the *right* are the associated Spearman’s rank correlation matrices of the environmental variables of corresponding samples. **(A–C)** Distance-based redundancy analysis (db-RDA) and Spearman’s rank correlation on the complete dataset **(A)**, the open-water sample subset **(B)**, and the under-ice sample subset **(C)**. The date of sampling corresponds to the last part of the sample name. Spearman’s rank correlation: ***p < 0.01, **p < 0.05, and *p < 0.1.

The db-RDA of all samples and available abiotic environmental data clearly showed the strong seasonality of the region and the response by the eukaryotic communities along the first axis (40.6% of the variance, significant *p* = 0.001) (Figure 3A), which was associated with gradients in temperature and ice cover. Greater ice thickness and higher TP concentrations were associated with the under-ice samples, whereas higher temperatures and higher TN/TP ratios (Figure 3A) were associated with the open-water samples. The TN/TP ratio was mainly driven by the higher TP concentrations under the ice compared to the open-water samples (Table 1). The sub-clusters in Figure 2, which were associated with lake size separated along the second axis, were consistent with the Bray–Curtis clustering. Overall, 14.5% of the variance was explained by the second axis, with DOC having the greatest contribution. Spearman’s rank correlation showed that the abiotic variables of the first axis—ice cover, temperature, and TP—were co-correlated. On the other hand, the explanatory variables of the second axis—DOC, conductivity, and TN—were co-correlated and negatively correlated with lake area.

To gain further insight, we repeated the analysis on the under-ice and open-water samples separately.

The db-RDA on the under-ice dataset (Figure 3B) showed that ice thickness was the most influential environmental variable along the first axis that separated the autumn and most of the spring under-ice samples. Spearman’s rank correlations showed a correlation between ice and TP. Conductivity, DOC, and TN were significantly correlated with each other.

The db-RDA carried out using only open-water samples (Figure 3C) was not significant. However, as seen in Figure 2, the larger lakes tended to separate from the smaller lakes along the first axis. Spearman’s rank correlations indicated significant negative correlations between lake area and conductivity, DOC, and TN. Significant positive correlations were seen among the same three variables (conductivity, DOC, and TN) (Figure 3C). The significance of individual variables was further examined by applying ANOVA to the open-water dataset, which indicated that conductivity (*p* = 0.055) was associated with lake size during the open-water season.

### Core Eukaryote Microbial Taxa

**FIGURE 4:**
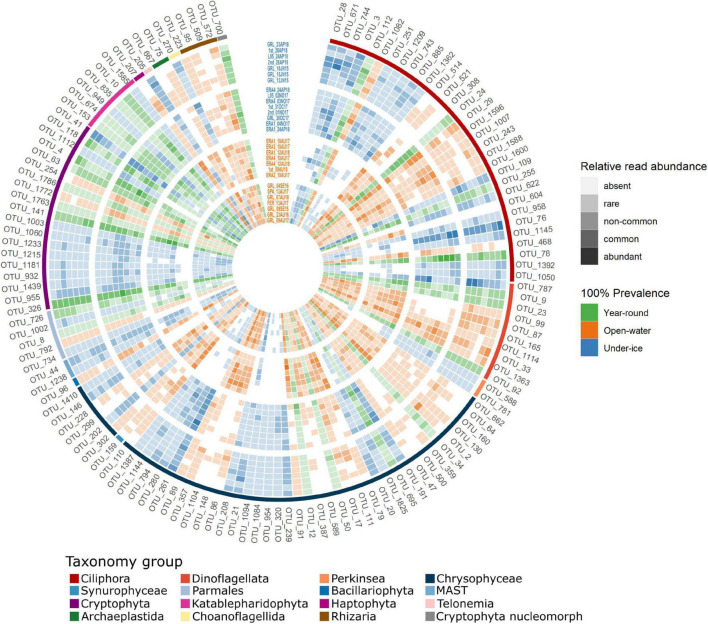
Heatmap of operational taxonomic units (OTUs) with 100% prevalence showing the relative read abundances of the core OTUs within all samples. Samples are arranged following the order of clustering in Figure 2, from the exterior to the center. Initial classification of OTUs is by prevalence. Those detected in all samples are shown in *shades of green*, open-water samples are in *shades of orange*, and under-ice samples in *shades of blue*. Intensity of the colors was based on the relative read abundance categories: absent (0), rare (<0.1%), non-common (0.1–1%), common (1–10%), and abundant (>10%). Taxonomy assignment is at the most precise taxonomic rank possible (see text). *Outside ring color* indicates the major taxonomic group of the OTUs.

We next identified potential core microbial eukaryotes within the geographic area of Greiner Lake. The OTUs that occurred in all (100%) of the under-ice or all (100%) of the open-water samples were defined as “core” OTUs. Using this definition, 133 putative core OTUs were identified (Figure 4). The core OTUs were primarily ciliates and dinoflagellates, but with high proportions of Chrysophyceae and Cryptophyceae. We verified the finer-level taxonomic assignment of these two groups using the Evolutionary Placement Algorithm (EPA). The 46 core Chrysophyceae OTUs were placed at 30 nodes. Globally, where there was a species- or genus-level taxonomic assignation by PR^2^, the EPA agreed (Supplementary Figure 2). For example, OTUs identified as *Dinobryon* were placed at nodes G, H, and I with verified *Dinobryon* spp. Similarly, OTUs classified as the genus *Kephyrion* were placed at nodes J and K with verified *Kephyrion* 18S rRNA sequences. Since *Ochromonas* is polyphyletic, OTUs that were affiliated to any *Ochromonas* in the NCBI taxonomy were named after the clade in which they appeared, in this case clade C-a (top part of the C clade) and clade C-b (bottom part of the C clade) (Figure 4 and Supplementary Figure 2). Similarly, the EPA placement separated cryptomonad species (Supplementary Figure 3) into various clades. The cryptophyte OTUs were placed into three separate clades, with the first clade consisting of an unresolved mix of named species of three genera: *Plagioselmis*, *Teleaulax*, and *Geminigera*. Since our OTUs were placed on a *Plagioselmis nannoplanctica* node, we designated these as *P. nannoplanctica*, which is described as a freshwater cryptophyte with the type species from Sweden (Novarino et al., 1994). The other two clades of cryptophytes were related to *Cryptomonas* species—*Cryptomonas ovata* and *Cryptomonas reflexa*—and a clade referred to in PR^2^ as “Basal Cryptophyceae” (Supplementary Figure 3). In addition, OTU_700, which occurred in all samples, was a cryptophyte nucleomorph and was significantly correlated with the ensemble of *P. nannoplanctica* OTUs (cor. > 0.7, *p* < 0.0001), suggesting that OTU 700 is the *P. nannoplanctica* nucleomorph.

### Seasonal Operational Taxonomic Units

**FIGURE 5:**
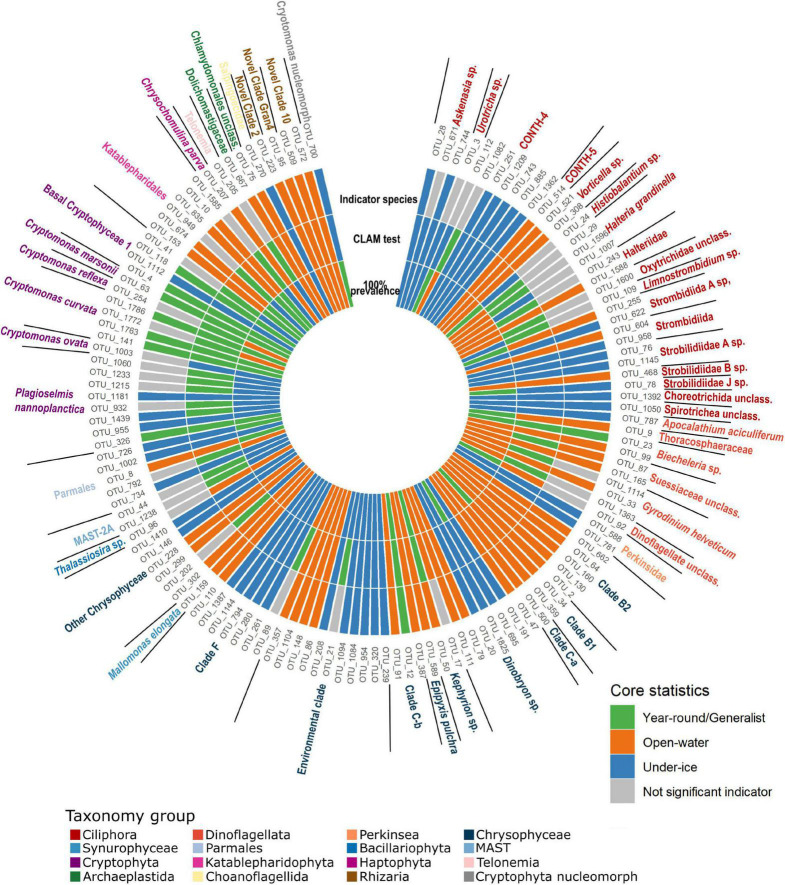
Core species categorizations. *Innermost ring* indicates initial classification from 100% prevalence data in all under ice, all open water, or both (from Figure 4). *Middle ring* shows the categorization of operational taxonomic units (OTUs) as under ice or open water or all year using the multinomial species classification method (CLAM test). *Outermost ring* shows the results of IndVal analysis showing the positive indicator OTUs for under-ice, open-water, or all-year conditions. Generalist OTUs, where the initial and CLAM tests agreed, are also marked in *green in the outer ring*. For all three rings, positive results are color coded, with year-round generalists in *green*, open-water OTUs in *orange*, and under-ice OTUs in *blue*. *Gray in the outermost ring* indicates OTUs that were not significant InVal indicators of any of the three conditions and differed between the CLAM and initial classifications.

There were 133 putative core OTUs, of which 25 OTUs were found in every sample and were provisionally considered “year-round” (Figure 4). After removing putative generalists, 55 “under-ice” and 53 “open-water” OTUs remained (Figure 5). The groups were provisionally designated generalists, under-ice, and open-water specialists (Figure 5, inner ring).

**FIGURE 6:**
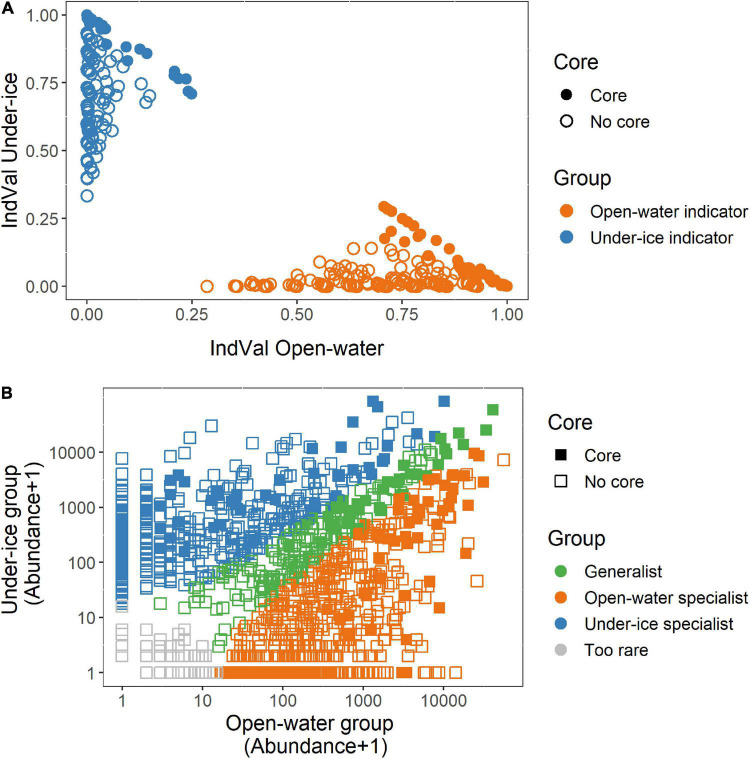
Open-water and under-ice statistical tests and classification. Tests were carried out on the entire dataset. *Filled symbols* are operational taxonomic units (OTUs) that were identified in the core analysis (Figures 4, 5) and *open symbols* are those not in the core. **(A)** Indicator (IndVal) OTUs comparing under ice (*blue symbols*) and open water (*orange symbols*). **(B)** Multinomial species classification method (CLAM) test signifying the open-water (*orange*), under-ice (*blue*), or year-round (*green*) specialists. OTUs that were too rare to classify are shown in *light gray*.

To provide statistical support for this classification and to identify under-ice “winter” or open-water “summer” season specialists and indicators, two independent tests were carried out. These tests involved analysis of all 1,435 microbial eukaryote OTUs from all samples. For this, we determined seasonal indicator OTUs using IndVal. Since some OTUs were assigned to the same species, we considered the OTUs with 98% similarity level as ecotypes. Out of the total, 582 OTUs were classified as significant (*p* ≤ 0.05) season indicators of one condition or the other. Of these, 337 were characteristic of the open-water samples and 245 were associated with under-ice samples (Figure 6A). Out of the 133 putative core OTUs, the IndVal tests placed 48 as open-water and 44 as under-ice indicators (Figure 5, outer ring). When the initial generalist classification was confirmed by the CLAM test (see below), we marked the OTU as a generalist indicator (Figure 5, green slices).

The CLAM test was used to classify the 1,435 OTUs as either specialists for a given season, generalists, or too rare to be classified. From this, 192 were classified as generalists, 421 OTUs were under-ice specialists, 713 OTUs were open-water specialists, and 109 OTUs were too rare to be classified (Figure 6B). Examination of the 133 OTUs classified as the putative core community revealed that 36 were generalists, 49 were open-water specialists, and 48 were under-ice specialists (Figure 5, middle ring).

A majority of the putative core community OTUs (63%) showed consistency in their initial classification as generalists or as open-water or under-ice specialists with the CLAM classification and were also indicator species of a particular season. There were only 12 OTUs (9%) where the IndVal and CLAM classifications were not consistent. CLAM analysis resulted in the displacement of 26 under-ice and open-water OTUs into the generalist category. Conversely, 15 members of the “all seasons” category were classified as specialists of a season (Figure 5). Only one of the OTUs (OTU_1007), which was a *Halteria*, switched between open water and under ice in the initial *vs.* the CLAM categories.

We then assessed the overlap of the “core taxa” and the taxa with the highest read proportions (top OTUs). We compared the 133 core OTUs (Figure 4) and the 70 top five OTUs (Supplementary Figure 1) from all lakes (Supplementary Figure 4). Only 27 OTUs were shared by the core and in the top five OTUs. At the major group level, ciliates were the most overrepresented in the top five compared to the core, while chrysophytes were the most overrepresented in the core compared to the top five OTUs. Some top OTUs were associated with the Bray–Curtis clustering according to a SIMPER analysis, with the 10 OTUs that best explained the difference between the open-water and under-ice clusters all among the top five OTUs (Supplementary Figure 1). Eight of the top OTUs separating the two clusters were also core species, as defined in our initial classification (Figures 4, 5). Eight were also classified as either open-water or under-ice indicators using IndVal (Supplementary Table 2). The SIMPER analysis was then applied to the separate under-ice and open-water sub-clusters. Overall, the 10 best explanatory OTUs from each comparison (under ice *vs*. open water, Ice1 *vs*. Ice2, and Open3 *vs*. Open4) accounted for 21.8, 30, and 21.7% of the difference between the respective compared groups (Supplementary Table 2).

## Discussion

### General Microbial Diversity

Given the direction of human-induced climate change, freshwater biodiversity is expected to be impacted by abiotic disturbance regimes and physical habitat modifications. The changes may disfavor existing resident taxa and favor invasions of taxa previously excluded by Arctic conditions (Wrona et al., 2006, 2016). However, basic knowledge of the core and characteristic taxa is required before short- or long-term impacts from ongoing global warming or changes in the precipitation patterns linked to recent anthropogenic climate perturbations can be assessed. Here, we identified the most common and widespread resident taxa living under ice and in open waters of small and large lakes of the Ekaluktutiak region of southern Victoria Island. Among the 133 OTUs that were found in either all open-water or all under-ice lakes, none were exclusive to a particular season (Figure 4), suggesting that the pool of phylotypes that always persist in most lakes may have been underestimated using our strict criteria of 100% prevalence in all lakes on all dates. Despite the ubiquity of many OTUS, the relative abundances of OTUs from the pool differed by lake and day of sampling (Figure 4). Targeted in-depth studies of these lakes are needed to fully understand successional patterns.

At the community level, the analysis showed the marked seasonality and the differences between the under-ice and open-water communities. In addition to the strong influence of the number of hours of daylight and temperature driving seasonality, communities were influenced by specific environmental conditions associated with lake ecology. Species succession is known for phytoplankton and has been reported for zooplankton in other Arctic lakes (Rautio et al., 2011b), but less is known about heterotrophic microbial eukaryotes in winter and summer communities. Annual production largely depends on inorganic nutrient supply in the summer, but in ice-covered lakes, organic matter produced in the summer is required to sustain biological activity during winter (Lizotte, 2009; Hampton et al., 2017; Teufel et al., 2017; de Melo et al., 2019). Dissolved organic matter (DOM) is processed by the winter bacteria that support heterotrophic flagellates, dinoflagellates, and ciliates (Lizotte, 2009; Vigneron et al., 2019), which are food for small zooplankton and which maintain resident fish populations (Grosbois et al., 2020), ensuring a healthy ecosystem.

To reveal the trends that might have been masked by the season, environmental factors associated with the open-water and under-ice communities were analyzed separately. The under-ice communities were separated along the principal axis by day length, ice thickness, and TP (Figure 3). The effects of day length and ice thickness were consistent with the succession of microbial eukaryotes over the winter, from freeze-up in October to when ice melts in June. The effect of TP could be associated with both biological and abiotic conditions over winter. TP is a key lake health indicator since lakes are often phosphorus-limited, but excess phosphorus can result in eutrophication and suitable conditions for harmful algal bloom (HAB) events. Recently, a naturally eutrophic lake with high TP levels was reported from near the four ERA lakes sampled here (Ayala-Borda et al., 2021). The high TP load was thought to be due to the phosphorus-rich bedrock in the immediate area combined with high snow accumulation due to local lake morphology and orientation, which resulted in the increased water tracks in early spring maintaining summer phytoplankton production. Here, we found that the TP concentrations were greater under the ice, in agreement with another recent study reporting higher winter inventories of TP (Imbeau et al., 2021). The generally lower TP concentrations in samples from August and September indicate that TP was drawn down over the growing season, and the low Chl *a* levels are in keeping with most of the lakes in the region being more oligotrophic.

In contrast to under-ice conditions, during the open-water season DOC and conductivity had the greatest effect on the communities. Both DOC and conductivity were influenced by lake area, with smaller lakes more affected by near-shore allochthonous DOC (Abnizova et al., 2014) and conductivity, which increases in the summer due to evaporation (Young and Abnizova, 2011; Andresen and Lougheed, 2015). The moderately lower conductivity in Greiner Lake in open- *vs.* under-ice conditions (Table 1) would be consistent with salt ejection during ice formation in the winter (Bluteau et al., 2017) and lake flushing during the spring thaw.

The SIMPER analysis indicated that the seasonal and within-season community clustering was associated with OTUs with high relative read abundance, with rare species having less individual weight. In particular, diverse almost lake-specific ciliates were dominant (Supplementary Figures 1, 4 and Supplementary Table 2). Although the high 18S rRNA gene copy numbers (Blazewicz et al., 2013) in ciliates and the large numbers of ribosomes (from cDNA) in these larger cells could explain some of their high relative read abundance, the diversity of ciliates in the top five OTUs of lakes could be consistent with the ephemeral blooms of individual species. The sporadic occurrences of different ciliate OTUs suggest that ciliate species could be sensitive indicators of local lake conditions, as previously suggested for the Antarctic Dry Valley communities (Xu et al., 2014). Most ciliates likely form cysts (Verni and Rosati, 2011), including Spirotrichea and species such as the *Halteria grandinella* (Foissner et al., 2007) found here. Encystment facilitates the dispersion of species (Verni and Rosati, 2011) and provides inoculum for population recruitment when conditions are suitable for a given species. Once a “bloom” is terminated, the local lake would be a repository for the locally favored ciliate taxa.

All chrysophytes are believed to produce cysts (stomatocysts or statospores) as a response to stresses (Holen, 2014). In the Ekaluktutiak region, chrysophyte OTUs tended to separate the open-water *vs.* under-ice samples, especially in the smaller ERA lakes. Our finding that different species of chrysophytes were seen under different conditions reinforces the use of chrysophytes as indicators of lake status. For example, chrysophyte cysts, which are species-specific, are used in paleolimnological studies as indicators of lake conductivity and area (Pla and Anderson, 2005).

### Mixotrophy in Arctic Lakes

The prevalence of mixotrophic taxa over both seasons and all lakes was striking. Mixotrophic microbial eukaryotes range from being primarily heterotrophic and using phototrophy as an energy supplement to being primarily phototrophic and using phagotrophy to supplement growth under low light and darkness (Lie et al., 2017). Mixotrophy is a useful strategy under low light conditions in the High Arctic (Charvet et al., 2014). Mixotrophs can also be classified as facultative or obligate by requiring prey to obtain essential nutrients for growth (Lie et al., 2018). In the core microbiome of Ekaluktutiak, we retrieved multiple mixotrophs from a range of phylogenetic groups and potential diverse behaviors. For example, several chloroplast-containing *Ochromonas* sp. are principally phagotrophic (Andersson et al., 1989; Lie et al., 2018), which may be the case for the core *Ochromonas* related to another Arctic ice-associated *Ochromonas* (CCMP2298). CCMP2298 is probably an obligate phagotroph as it lacks inorganic nitrogen transporters and would only be able to obtain nitrogen from prey (Terrado et al., 2015). Other chrysophytes such as *Dinobryon* can supplement their growth with prey ingestion under low light conditions (Caron et al., 1993), consistent with being found during the ice-covered season. *Uroglena americana* is an example of an obligate phagotroph using prey to obtain growth factors (Sanders, 2011; Kazamia et al., 2012).

The cryptophytes *P. nannoplanctica* and *Cryptomonas curvata*, both of which were found in the under-ice core, ingest bacteria to acquire nutrients (P and N) when they are depleted in the environment (Izaguirre et al., 2012), but also use phagotrophy to maintain populations under low light regimes (Mitra et al., 2016). Their predominance in the under-ice core suggests the latter as a primary driver in our Arctic lakes. Another way of being mixotrophic is to acquire phototrophy by endosymbiosis and kleptoplasty (Stoecker et al., 2009). *Halteria*, *Askenasia*, *Vorticella*, *Limnostrombidium*, and *Histiobalantium* (Figure 5) are all reported to be kleptoplastidic (Stoecker et al., 2009; Esteban et al., 2010). Overall, mixotrophic taxa, whether obligate or optional, appeared to be favored, at least during the times of year we were able to sample. The high zooplankton populations in the lakes at both times of year (Grosbois et al., 2022) would also benefit since, at least in North American boreal lakes, zooplankton show a preference for mixotrophic species (Hansson et al., 2019).

### All-Year Generalists

Most of the generalist core taxa were cryptophytes, with one chrysophyte, the two lineages that are typical of northern temperate (Cruaud et al., 2019) and polar freshwater environments (Charvet et al., 2012b). The majority of the generalist core OTUs belonged to mixotrophic–photosynthetic cryptophyte lineages. Their ability to survive under ice and in open water could be aided by a capacity to adjust their pigment profiles for high or low light conditions, as reported for the marine Arctic cryptomonad *Baffinella frigidus* (Daugbjerg et al., 2018). However, this remains to be verified for these freshwater species, and the adaptability to light could be a more general feature of cryptomonads (Greenwold et al., 2019). Two other cryptomonads were in the basal group of Cryptophyceae (Supplementary Figure 3). The nearest known relatives of this environmental cryptomonad clade are the non-photosynthetic Goniomonadales suggesting that the OTUs in this study may be purely heterotrophic. The two other year-round core taxa were from probable mixotrophic lineages: clade Cb Chrysophyceae (Supplementary Figure 3) and the dinoflagellate *Biecheleria* (Suessiaceae). Freshwater members of *Biecheleria* have been previously classified as *Woloszynskia pseudopalustris* (Moestrup et al., 2009). *Biecheleria* are typical of most photosynthetic woloszynskioid dinoflagellates and most likely mixotrophic, making the majority of the year-round “generalist” species able to photosynthesize when light is available and switch to heterotrophy under low light or darkness.

### Under Ice

The under-ice specialists were more trophically diverse compared to the year-round generalist core group. The phylogenetically diverse taxa included phagotrophic mixotrophs, parasites, and strict heterotrophs. Specifically, within the under-ice specialist category were 17 likely mixotrophic stramenopiles, mostly within the Chrysophyceae. Chrysophyceae included mixotrophic genera belonging to *Dinobryon* spp. and OTUs at the nodes of the freshwater genera *Chromophyton* and *Chrysosaccu*s spp. Putative heterotrophic chrysophytes included *Paraphysomonas* spp. and other unclassified Chrysophyceae (Supplementary Figure 2), with unknown trophic status. Several Parmales, an order that is synonymous with Bolidophyceae, which are primarily marine but reported from Lake Baikal (Annenkova et al., 2020), were also among the under-ice indicators. The flagellated stages of Parmales are thought to be mixotrophic (Frias-Lopez et al., 2009). Two cryptophyte under-ice OTUs were assigned to *P. nannoplanctica*, which is presumably mixotrophic. Recent taxonomic revisions have suggested that *Plagioselmis* may be part of a di-morphospecies duo related to *Teleaulax* (Altenburger et al., 2020), which have complex life stages and serve as photosynthetic partners in some ciliates, suggesting potential for even more complex trophic roles of some under-ice taxa.

Alveolates that were under-ice specialists included parasitic Perkinsideae and ciliates classified within the well-supported 18S rRNA CONThree (CONTH) clade. The CONTH clade is named after the three major ciliate groups that were initially placed in the clade: Colpodea, Oligohymenophorea, and nassophoreans. CONTH now includes Phyllopharyngea and prosomateans (see UniEuk taxonomy^1^). The PR2 database places the environmental clade CONTH-4 into the CONTH clade. CONTH-4 appears to be related to the parasitic *Cryptocaryon irritans*, which is a Prostomatea that infects fish (Wright and Colorni, 2002; Yambot et al., 2003).

Heterotrophic ciliates under ice were diverse and included *Askenasia*, other Prostomatea, and various Spirotrichea. Also in the under-ice group were several dinoflagellates including a putative mixotrophic dinoflagellate classified as *Apocalathium aciculiferum*. The only other representative of a major group consistently categorized as under ice was a choanoflagellate in the Salpingoecidae family, which are bacterivores. The diverse mixotrophic and heterotrophic microorganisms would be essential for maintaining active overwintering zooplankton populations (Rautio et al., 2011b).

### Open Water

Open-water communities included more photosynthetic species, with two green algal OTUs, Chlamydomonadales sp. and Dolichomastigaceae, and a diatom. Other photosynthetic core OTUs were from mixotrophic lineages: *Dinobryon* spp., an undescribed ochromonad, and two other chrysophytes, *Uroglena* and *Mallomonas elongata*, which are potential bloom-forming species. We also found a second *Biecheleria* OTU, other unclassified Suessiaceae, and the mixotrophic haptophyte *Chrysochromulina parva*. Majority of the heterotrophic open-water core OTUs were alveolates; among these were ciliates classified in PR^2^ in the group CONTH-5, also distantly related to the fish parasite (Yambot et al., 2003), the genus *Vorticella* and other ciliate grazers from the class Spirotrichea. One putatively heterotroph dinoflagellate, *Gyrodinium helveticum*, was also indicative of open water along with the presumed non-photosynthetic chrysophyte *Paraphysomonas*, Katablepharidales, and Cercozoa.

### Seasonal Ecotypes

This analysis highlighted closely related OTUs with different seasonal preferences. Among these were clades that were almost always open-water specialists, such as chrysophyte clades B1 and B2 (Figure 5) and others that were almost always associated with under-ice conditions, such as the ciliates classified as *Askenasia*, *Urotricha*, and the CONTH-4 group. Other OTUs that were nominally the same species or genus were found in both the open-water and under-ice core groups, sometimes with a generalist representative, suggesting ecotype diversity and specialization in the same or closely related species. For example, *Dinobryon* spp. included both open-water and under-ice OTUs, and the closely related clade F chrysophytes were predominantly under ice, but with a single more open-water-trending OTU. Other species showed some seasonal preference, but were also found year-round in all lakes. In particular, the cryptophyte *P. nannoplanctica* was diverse with 9 OTUs found in all lakes, but with a tendency to occur in winter. In contrast, the dinoflagellate *Biecheleria* and the chrysophytes in clade C-b were trending toward a summer preference, but with one OTU found year-round (Figure 5 and Supplementary Figure 2) for each. Similar seasonal diversity has been reported in the diatom *Ditylum brightwellii* (Koester et al., 2010) and geographic diversity described for strains of *Prymnesium parvum* (Lysgaard et al., 2018), which suggests that considerable genetic or potentially epigenetic diversity (Ait-Mohamed et al., 2020) within species is widespread. This environmental microdiversity suggests caution when interpreting laboratory-based experiments on single strains of diverse lineages.

### Implications for Human Activities

Some OTUs retrieved in the core could potentially have harmful impacts or have impacts on freshwater services. For example, OTUs related to *U. americana*, a colonial flagellate, were retrieved in the open-water samples. This species causes a distinct unpleasant odor in drinking water (Juttner, 1995), and *U. americana* blooms, although not toxic, impacts local human populations. The presence of potential HABs emphasizes a need for monitoring lakes that are used by local communities. Other HABs are harmful to the health of the lake ecosystem. For example, the dinoflagellate *A. aciculiferum*, previously known as *Peridinium aciculiferum* (Craveiro et al., 2017), was reported to form high biomass under the ice in Swedish lakes and can produce a toxin under P limitation. This toxin can be lethal for other phytoplankton and is linked to poor recruitment of larvae of the salmonid whitefish *Coregonus albula* (Rengefors and Legrand, 2001). In this context, the relative abundance of reads classified as *A*. *aciculiferum* (OTU_787) was high in the under-ice samples collected in June 2015 (GRL_10JU15, GRL_15JU15, and GRL_12JU15) and the end of October 2017 (GRL_30OC17) from Greiner Lake (Figure 4), suggesting potential for a bloom. The effect on other phytoplankton or whitefish that live in the lake has not been studied, but would warrant further investigation.

## Conclusion

We investigated both under-ice and open-water communities from shallow ponds to the 588-km^2^ Tahiryuaq, which is an important habitat for sea-run Arctic char and local commercial fisheries. Core microbial taxa were identified, with year-round occurrences of presumably mixotrophic Cryptophyceae, a chrysophyte, and a dinoflagellate. Seasonally defined core taxa included parasites (Perkinsidae and the CONTH ciliates), heterotrophs (other ciliates, dinoflagellates, colorless chrysophytes, katablepharids, Cercozoa, and choanoflagellates) and mixotrophs (cryptophytes, chrysophytes, dinoflagellates, and kleptoplastidic ciliates). The core OTUs were not synonymous with the five most abundant OTUs from each sample. Ciliates made up nearly a quarter of the open-water and under-ice core OTUs, but accounted for over two-thirds of the reads of the top five most abundant OTUs of the individual samples, suggesting the potential utility of monitoring ciliate ecotypes for changes in lake ecology. Our survey of the dominant and most common species or ecotypes presently inhabiting these Arctic water bodies provides context for future studies.

### Impact Statement

Microbial eukaryotes support the upper trophic levels providing food to zooplankton, which are food for fish, a primary staple and cultural icon for local Inuvialuit. This study establishes the first overview of the microbial eukaryote freshwater communities on Victoria Island, in small and large lakes that are threatened by ongoing climate warming.

## Data Availability Statement

The datasets presented in this study can be found in online repositories. The names of the repository/repositories and accession number(s) can be found in the article/Supplementary Material.

## Author Contributions

CL and MR conceived of the study and collected samples. MP and CL wrote the manuscript with input from MR. MP carried out laboratory work, bioinformatic analysis, and drafted figures. MR provided ancillary data and map. All authors contributed to the article and approved the submitted version.

## Conflict of Interest

The authors declare that the research was conducted in the absence of any commercial or financial relationships that could be construed as a potential conflict of interest.

## Publisher’s Note

All claims expressed in this article are solely those of the authors and do not necessarily represent those of their affiliated organizations, or those of the publisher, the editors and the reviewers. Any product that may be evaluated in this article, or claim that may be made by its manufacturer, is not guaranteed or endorsed by the publisher.

## Footnotes

1
https://eukmap.unieuk.org/taxonomy/1163
