# Supplementary material for: Freshwater Microbial Eukaryotic Core Communities, Open-Water and Under-Ice Specialists in Southern Victoria Island Lakes (Ekaluktutiak, NU, Canada)
Source: Front Microbiol. 2022 Feb 11;12:786094. doi: 10.3389/fmicb.2021.786094 (PMC8873588; doi:10.3389/fmicb.2021.786094)
Supplement: Supplementary file 3 [file Data_Sheet_3.PDF]

Supplementary Tables S1 and S2

Freshwater microbial eukaryotic core communities, open-water and under-ice specialists in southern Victoria Island lakes (Ekaluktutiak, Nunavut, Canada)

Marianne Potvin[1], Milla Rautio[2,3,4], Connie Lovejoy[1]

[1] Département de biologie, Québec Océan, and Institut intégrative et des systèmes (IBIS) Université Laval, Québec, QC, Canada.

[2]Département des sciences fondamentales, Université du Québec à Chicoutimi, Chicoutimi, QC, Canada

[3]GRIL – Groupe de Recherche Interuniversitaire de Limnologie, Montréal, QC, Canada.

[4] Centre d'études nordiques (CEN), Québec, QC, Canada

Supplementary Table S1: Read results of from the pooled libraries of individual lakes sampled on a given day. Lake sample abbreviations as in Figure 1. The order of the in the table follows the Bray-Curtis clustering from Figure 2. Three to ten separate libraries were generated for each date-lake combination using available samples. . Raw reads (reads retrieved from Illumina MiSeq platform), filtered reads (merged reads that passed quality filters and chimera removal), final reads (non-targeted reads removed). Source of nucleic acids (RNA or DNA) followed by size fraction as a subscript (L or S for large or small). In 2015 additional depth samples were collected from Grenier lake and the depth for these samples given as the leading 2 place (0, 4 or 8 m).

Sample	Lake	Date	Raw reads	Filtered reads	Final reads	Libraries
**GRL_23AP18**	Greiner	2018-04-23	169188	66957	65984	RNA, RNA, DNA, DNA
**L05_24AP18**	L05	2018-04-24	182232	81205	77094	RNA, RNA, DNA, DNA
**1ST_26AP18**	1st lake	2018-04-26	195434	83565	83275	RNA, RNA, DNA, DNA
**2ND_26AP18**	2nd lake	2018-04-26	254103	122450	121962	RNA, RNA RNA[1], DNA, DNA
**GR_10JN15**	Greiner	2015-06-10	277626	142865	142338	0mRNA, 0mDNA, 0mDNA, 4mRNA, 4mDNA, 4mDNA, 8mDNA
**GR_15JN15**	Greiner	2015-06-15	342399	178169	177569	0mRNA, 0mDNA, 0mRNA, 0mDNA, 4mRNA, 4mDNA, 4mDNA, 8mDNA, 8mDNA
**GR_12JN15**	Greiner	2015-06-12	190787	102167	97777	RNA, DNA, RNA, DNA, DNA
**ERA4_24AP18**	ERA 4	2018-04-24	181445	79337	69980	RNA, RNA, DNA, DNA
**L05_02NO17**	L05	2017-11-02	181884	88897	88542	RNA, RNA, DNA, DNA
**ERA4_03NO17**	ERA 4	2017-11-03	192221	98145	97646	RNA, RNA, DNA, DNA
**2ND_01NO17**	2nd lake	2017-11-01	204277	101428	100942	RNA, RNA, DNA, DNA
**1ST_31OC17**	1st lake	2017-10-31	187219	91952	78596	RNA, RNA, DNA, DNA
**GR_30OC17**	Greiner	2017-10-30	168064	75216	73202	RNA, RNA, DNA, DNA
**ERA1_04NO17**	ERA 1	2017-11-04	205360	100402	90073	RNA, RNA, DNA, DNA
**ERA1_24AP18**	ERA 1	2018-04-24	178606	73753	68529	RNA, RNA, DNA, DNA
**ERA1_10AU17**	ERA 1	2017-08-10	138565	57605	52913	RNA, DNA, DNA
**ERA3_10AU17**	ERA 3	2017-08-10	243402	123280	110782	RNA, RNA, DNA, DNA
**ERA1_12AU18**	ERA 1	2018-08-12	162431	80428	77803	RNA, DNA, DNA
**ERA4_10AU17**	ERA 4	2017-08-10	188516	90045	82175	RNA, RNA, DNA, DNA
**ERA4_12AU18**	ERA 4	2018-08-12	160603	78529	36839	RNA, DNA, DNA
**1ST_09AU18**	1st lake	2018-08-09	132918	60932	57974	RNA, DNA, DNA
**ERA2_10AU17**	ERA 2	2017-08-10	177064	90003	86672	RNA, RNA, DNA, DNA
**GR_04SE16**	Greiner	2016-09-04	173067	99416	86395	RNA, DNA, RNA
**SPA_13AU17**	Spawning	2017-08-13	193355	99493	96225	RNA, RNA, DNA, DNA
**GR_07AU18**	Greiner	2018-08-07	162707	85403	76954	RNA, DNA, DNA
**FER_13AU17**	Tahiryuaq	2018-08-13	199351	86705	83700	RNA, RNA, DNA, DNA
**GR_09SE15**	Greiner	2015-09-09	420660	197766	179413	0mRNA, 0mDNA, 0mDNA, 4mRNA, 4mDNA, 4mRNA, 4mDNA, 8mRNA, 8mDNA, 8mDNA
**GR_23AU16**	Greiner	2016-08-23	178565	86317	83439	RNA, DNA, RNA, DNA
**GR_09AU17**	Greiner	2017-08-09	440860	190419	172748	RNALrep1, RNASrep1, DNALrep1, DNASrep1, RNALrep2, RNASrep2, DNALrep2, DNASrep2

[1] There were 2 replicate filters collected and processed separately.
[2] An additional non size fractionated sample (total DNA) was collected and processed separately
[3] For some samples, not all extracted DNA could be amplified and only 3 source libraries were available.

Supplementary Table S2: Top 10 contributor OTUs to SIMPER results. Contribution to explanatory value (contr) and standard deviation (sd) and ratio is contribution divided by the standard deviation.   The average abundance per group a (ava) and group b (avb). Cumulative sum of the explanatory contribution (cumsum), significance value (*p*). Taxonomy from group (Group Figure 4, the core classifications are from Figure 5: Year-round/Generalist (green), Open-water (orange), Under-ice (blue), no core/not significant indicator (grey). Colors under Group correspond to taxonomic color codes in Figures 5.

Comparison	***OTU_ID***	***contr***	***sd***	***ratio***	***ava***	***avb***	***cumsum***	***p***	***Taxonomy***	***Group***	***100%***	***CLAM***	***IndVal***
Open water versus Under ice	*OTU_76*	*0.031*	*0.029*	*1.08*	*0.063*	*0.001*	*0.036*	*0.0099*	*Strobilidiidae A*				
	OTU_78	*0.028*	*0.045*	*0.62*	*0.059*	*0.008*	*0.069*	*0.0891*	*Strobilidiidae J*				
	OTU_787	*0.021*	*0.041*	*0.52*	*0.043*	*0.001*	*0.094*	*0.0693*	*Apocalathium aciculiferum*				
	OTU_642	*0.021*	*0.028*	*0.74*	*0.006*	*0.042*	*0.118*	*0.0495*	*Litostomatea XXX sp.*				
	OTU_141	*0.019*	*0.032*	*0.60*	*0.018*	*0.038*	*0.140*	*0.2079*	*Cryptomonas curvata*				
	OTU_370	*0.016*	*0.029*	*0.56*	*0.032*	*0.003*	*0.159*	*0.2376*	*Vorticella unclassified*				
	OTU_326	0.015	0.012	1.25	0.041	0.029	0.177	0.5545	*Pelagioselmis*				
	OTU_261	0.014	0.014	0.98	0.028	0.000	0.193	0.0099	Chrysophyceae Clade-F				
	OTU_2	0.011	0.011	0.99	0.001	0.023	0.205	0.0099	Chrysophyceae XXX sp.				
	OTU_33	0.011	0.012	0.91	0.006	0.024	0.218	0.0198	*Gyrodinium helveticum*				
Ice 1 versus Ice 2	OTU_78	0.045	0.055	0.82	0.016	0.096	0.057	0.0198	Strobilidiidae J				
	OTU_787	0.036	0.050	0.71	0.064	0.025	0.102	0.0495	*Apocalathium aciculiferum*				
	OTU_76	0.032	0.025	1.27	0.056	0.070	0.142	0.1683	Strobilidiidae A				
	OTU_370	0.027	0.033	0.80	0.018	0.044	0.176	0.0594	*Vorticella* unclassified				
	OTU_554	0.020	0.017	1.19	0.041	0.000	0.202	0.0099	Isochrysidales unclassified				
	OTU_261	0.019	0.014	1.38	0.008	0.045	0.226	0.0099	Chrysophyceae Clade-F				
	OTU_55	0.015	0.015	1.02	0.032	0.006	0.245	0.0099	*Borghiella tenuissima*				
	OTU_326	0.015	0.013	1.21	0.041	0.041	0.265	0.4257	*Pelagioselmis*				
	OTU_4	0.014	0.015	0.93	0.004	0.031	0.283	0.0297	Basal Cryptophyceae-1 X				
	OTU_24	0.014	0.014	0.97	0.001	0.028	0.300	0.0099	*Histiobalantium* sp.				
Open 3 Versus Open 4	OTU_141	0.031	0.044	0.70	0.067	0.008	0.039	0.0990	*Cryptomonas curvata*				
	OTU_642	0.029	0.030	0.95	0.045	0.038	0.076	0.1485	Litostomatea XXX sp.				
	OTU_184	0.017	0.026	0.66	0.033	0.006	0.099	0.0891	Chrysophyceae X				
	OTU_326	0.016	0.010	1.59	0.016	0.042	0.119	0.5050	*Pelagioselmis*				
	OTU_33	0.014	0.012	1.13	0.025	0.022	0.136	0.0792	*Gyrodinium helveticum*				
	OTU_91	0.013	0.024	0.56	0.029	0.008	0.153	0.1782	Chrysophyceae Clade-C				
	OTU_553	0.013	0.033	0.41	0.000	0.026	0.170	0.0297	Ciliophora unclassified				
	OTU_70	0.013	0.027	0.47	0.026	0.002	0.187	0.0594	*Urosolenia eriensis*				
	OTU_2	0.012	0.011	1.14	0.020	0.026	0.202	0.1485	Chrysophyceae XXX sp.				
	OTU_68	0.012	0.016	0.74	0.024	0.001	0.217	0.0099	*Prorocentrum* sp.				
