# Supplementary material for: Freshwater Microbial Eukaryotic Core Communities, Open-Water and Under-Ice Specialists in Southern Victoria Island Lakes (Ekaluktutiak, NU, Canada)
Source: Front Microbiol. 2022 Feb 11;12:786094. doi: 10.3389/fmicb.2021.786094 (PMC8873588; doi:10.3389/fmicb.2021.786094)
Supplement: Supplementary file 4 [file Data_Sheet_4.PDF]

Supplementary Figures: S1, S2, S3, S4

Freshwater microbial eukaryotic core communities, open-water and under-ice specialists in southern Victoria Island lakes (Ekaluktutiak, Nunavut, Canada)

Marianne Potvin[1], Milla Rautio[2,3,4], Connie Lovejoy[1]

[1] Département de biologie, Québec Océan, and Institut intégrative et des systèmes (IBIS) Université Laval, Québec, QC, Canada.
[2] Département des sciences fondamentales, Université du Québec à Chicoutimi, Chicoutimi, QC, Canada
[3] GRIL – Groupe de Recherche Interuniversitaire de Limnologie, Montréal, QC, Canada.
[4] Centre d'études nordiques (CEN), Québec, QC, Canada

Supplementary Figure S1.

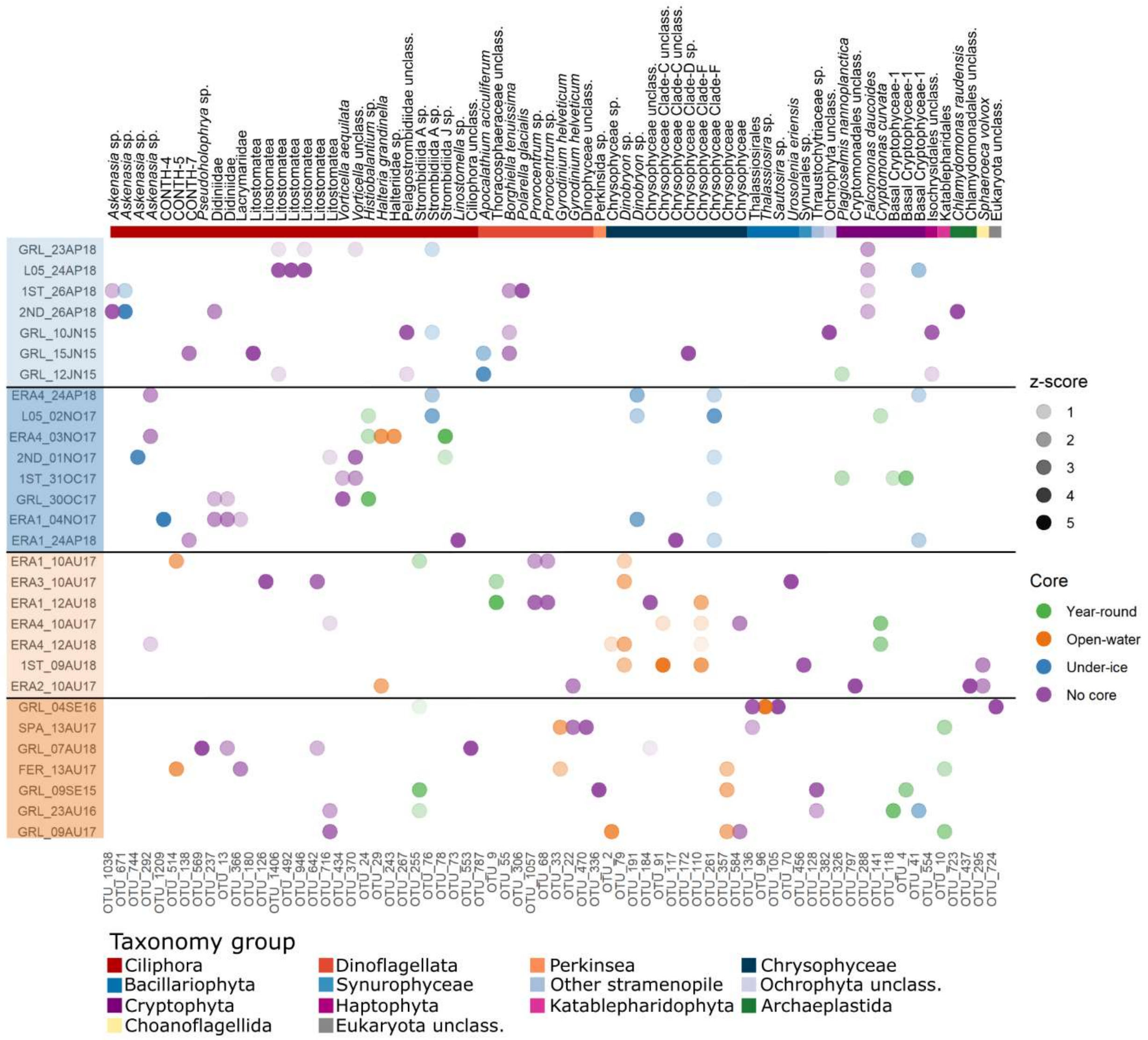

Supplementary Figure 1: Five most abundant OTUs retrieved in each sample arranged by major taxonomic group (top color bar) following color codes from Figure 4. The color of the dots corresponds to membership of the Figure 4 core (orange, blue, green) or no core categories (purple). Annual (green) OTUs detected in all samples, open-water (orange) OTUs detected in all open-water samples and under-ice (blue) OTUs detected in all under-ice samples. The intensity of shading indicates the z score, which is the number of standard deviations above the mean relative abundance for all OTUs.

Supplementary Figure S2.

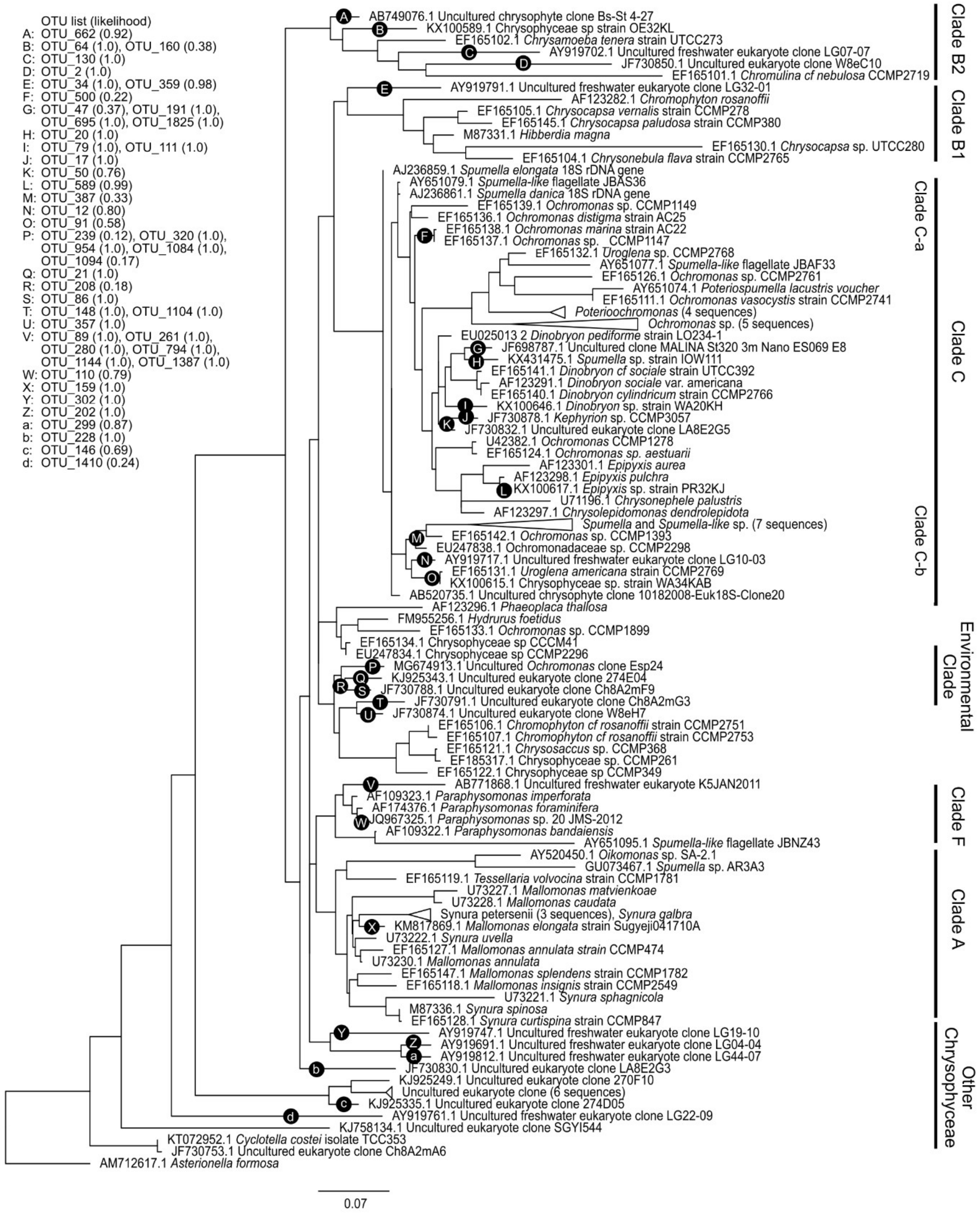

Supplementary Figure S2: Maximum likelihood tree: Chrysophyta with results from evolutionary placement algorithm (EPA) of core Chrysophyta OTUs. Core OTUs (Figure 4) were placed at nodes with the highest likelihood. OTUs from each of the indicated nodes are

listed on the right, with the EPA likelihood in parenthesis.
Supplementary Figure S3.

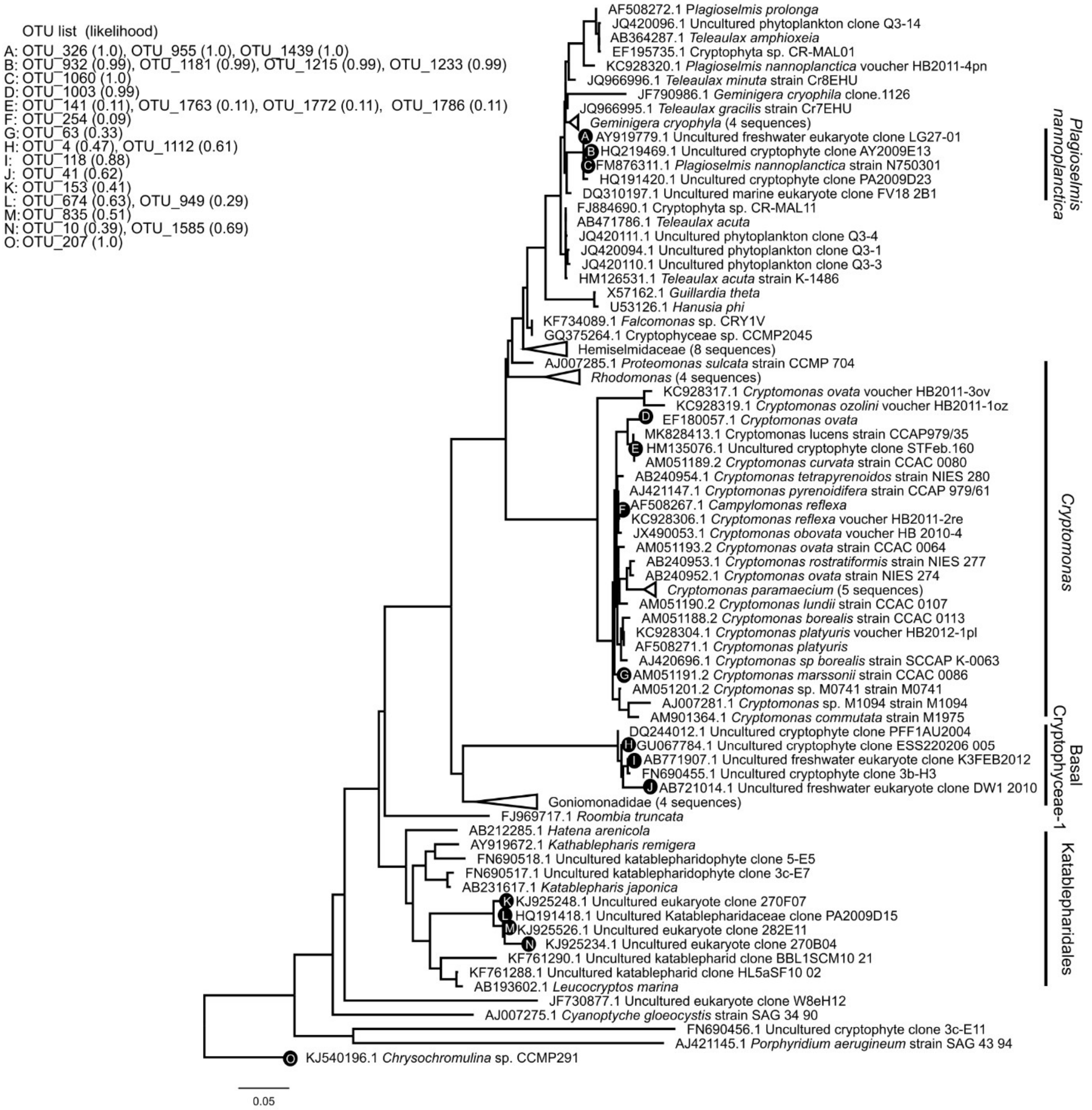

Supplementary Figure S3: Maximum likelihood tree: Cryptophyta and Katablepharidales with results from evolutionary placement algorithm (EPA). Core OTUs (Figure 4) were placed at nodes with the highest likelihood. OTUs from each of the indicated nodes are listed on the right, with the EPA likelihood in parenthesis.

Supplementary Figure S4.

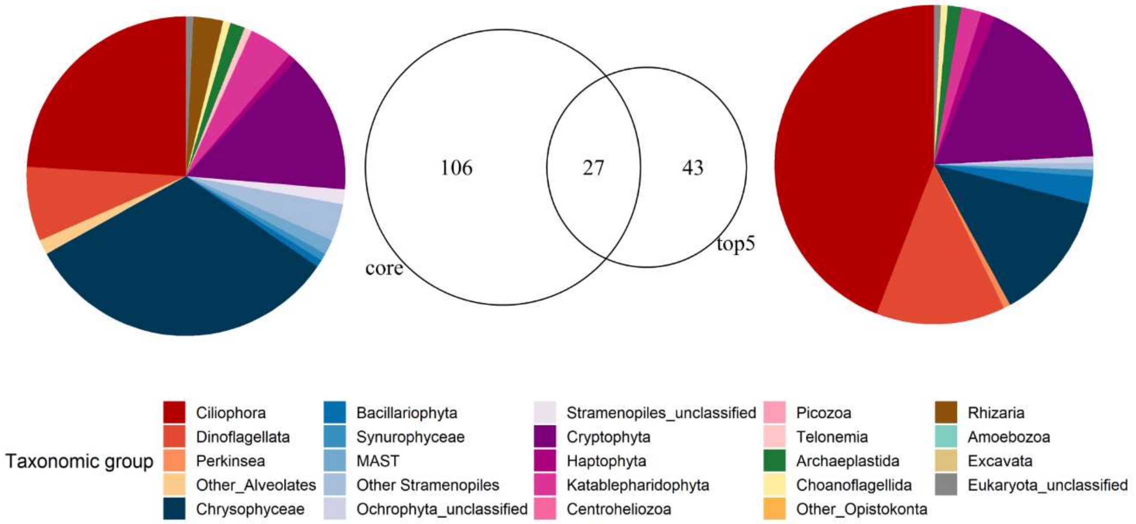

Supplementary Figure S4: Comparison od OTUs identified as core and top-five OTUs from each sample. Venn diagram indicates the number of OTUs from each category, with core OTUs on the right and top 5 OTUs on the left, with 27 OTUs shared. Pie charts show the proportion of OTUs assigned to each taxonomic group.
